# Barrier-Forming Potential of Epithelial Cells from the Exstrophic Bladder

**DOI:** 10.1016/j.ajpath.2022.03.009

**Published:** 2022-06

**Authors:** Jennifer Hinley, Rosalind Duke, Jessica Jinks, Jens Stahlschmidt, David Keene, Raimondo M. Cervellione, Imran Mushtaq, Paolo De Coppi, Massimo Garriboli, Jennifer Southgate

**Affiliations:** ∗ Jack Birch Unit for Molecular Carcinogenesis, Department of Biology and York Biomedical Research Institute, University of York, York, United Kingdom; † Department of Histopathology, St James's University Hospital, Leeds, United Kingdom; ‡ Department of Paediatric Urology, Royal Manchester Children's Hospital, Manchester, United Kingdom; § Department of Paediatric Urology, Great Ormond Street Hospital for Children National Health Service Trust, London, United Kingdom; ¶ National Institute for Health and Care Research Biomedical Research Centre, University College London Great Ormond Street Institute of Child Health, London, United Kingdom; ‖ Paediatric Urology, Evelina London Children's Hospital, London, United Kingdom

## Abstract

Bladder exstrophy (BEX) is a rare developmental abnormality resulting in an open, exposed bladder plate. Although normal bladder urothelium is a mitotically quiescent barrier epithelium, histologic studies of BEX epithelia report squamous and proliferative changes that can persist beyond surgical closure. The current study examined whether patient-derived BEX epithelial cells *in vitro* were capable of generating a barrier-forming epithelium under permissive conditions. Epithelial cells isolated from 11 BEX samples, classified histologically as transitional (*n* = 6) or squamous (*n* = 5), were propagated *in vitro*. In conditions conducive to differentiated tight barrier formation by normal human urothelial cell cultures, 8 of 11 BEX lines developed transepithelial electrical resistances of more than 1000 Ω.cm^2^, with 3 squamous lines failing to generate tight barriers. An inverse relationship was found between expression of squamous *KRT14* transcript and barrier development. Transcriptional drivers of urothelial differentiation *PPARG*, *GATA3*, and *FOXA1* showed reduced expression in squamous BEX cultures. These findings implicate developmental interruption of urothelial transcriptional programming in the spectrum of transitional to squamous epithelial phenotypes found in BEX. Assessment of BEX epithelial phenotype may inform management and treatment strategies, for which distinction between reversible versus intractably squamous epithelium could identify patients at risk of medical complications or those who are most appropriate for reconstructive tissue engineering strategies.

The bladder exstrophy–epispadias complex (BEEC) is a series of congenital genitourinary tract malformations resulting from failed partitioning of the cloacal membrane during midline closure. The BEEC spectrum ranges in severity from epispadias, characterized by failure of urethral closure alone, through classic bladder exstrophy (BEX), which results in an exposed bladder plate, to the most severe cloacal exstrophy associated with omphalocele, anal and vertebral defects [part of the complex of omphalocele, cloacal exstrophy, imperforate anus, and spinal defects (OEIS complex)]. The overall reported prevalence of BEEC is 5.2 per 100,000 fetuses, stillbirths, and live births, and is two to three times more prevalent in males than in females.^1^^,^^2^

The precise etiology of BEEC is unknown, although concordance rate studies suggest a predominantly genetic cause.^3^ The N-terminal truncated isoform of tumor protein p63, ΔNp63, has been linked to early bladder development in mice, in which expression of ΔNp63 occurs preferentially in the ventral bladder urothelium and p63^-/-^ knock-out mice present with a classic BEX phenotype.^4^ Fetal studies have suggested that the alternatively spliced isoform of p63 with transactivation domain TAp63α is the first detectable isoform in murine urothelial development, but by birth expression is replaced by ΔNp63, which acts in a dominant-negative manner to suppress TAp63 isoforms.^5^ In one human study, tissue-specific dysregulation of p63 isoforms was observed in 11 of 15 neonates with BEEC; however, no *TP63* gene mutations were present in the cohort.^6^ A genetic basis underpinning abnormal p63 expression in BEEC remains unconfirmed. Duplications of 22q11.2 have also been significantly linked to individuals with BEEC; however, no relevant gene has been identified to account for the pathogenesis of BEEC.^7^

The majority of BEEC patients undergo surgical reconstruction to achieve urinary continence and preserve renal function. Some centers favor closure of the bladder and abdominal wall shortly after birth,^8^ while others delay the primary closure to allow for growth of the typically small bladder plate.^9^ Depending on each center's approach, patients undergo further reconstructive surgery after the primary closure, generating the possibility to biopsy the bladder at three different time points: primary neonatal closure, delayed primary closure, and secondary surgery. Despite reconstructive or diversion surgery, long-term issues include lower urinary tract symptoms and a 700-fold increased risk of urothelial cancer.^10^

The lining of the normal bladder is a transitional epithelium known as urothelium, which is specialized to function as a tight urinary barrier to enable safe urine containment. Histologic studies of BEX urothelia have reported squamous metaplasia, inflammation, and proliferative changes, with loss of archetypical transitional differentiation markers including the superficial cell marker uroplakin 3a, the tight junction marker claudin 4, and transitional-type cytokeratin (CK)13 and CK20.^11^ Such observations led the authors to question whether urothelial differentiation would resolve after primary bladder closure. However, in a follow-up study the authors reported chronic inflammation to be present in “virtually all” secondary BEX samples, with persistence of squamous metaplasia and attendant proliferative changes.^12^ A recent study reported higher rates of squamous metaplasia and reduced expression of uroplakin 2 in delayed versus newborn bladder closure BEEC patients,^13^ suggesting that the timing of bladder closure may influence the urothelial phenotype. The underpinning question remains whether the phenotype of the BEX bladder epithelium is inherently altered by genetics, is affected by anomalous instructive (epigenetic) signaling during development, or is secondarily modulated by the abnormal open bladder environment. This knowledge would help inform current and future strategies for BEX management, including conventional reconstructive surgery, as well as the potential of using *in vitro*–expanded BEX epithelia in tissue-engineering approaches.

One experimental strategy to address the primary nature of BEX epithelium is *in vitro* cell culture, in which environment and extrinsic factors can be tightly controlled. Normal human urothelial (NHU) cells maintained as finite cell lines in growth factor–supplemented, serum-free, low-calcium (0.09 mmol/L) medium, acquire a proliferative CK13^-^/CK14^+^ squamous phenotype.^14^ Upon transfer to medium supplemented with bovine serum and near-physiological (2 mmol/L) calcium, NHU cells switch from squamous to transitional, becoming CK13^+^/CK14^−^ and developing functional tight barrier properties [transepithelial electrical resistance (TEER) >1000 Ω.cm^2^].^15^ Underpinning this differentiation is a network of nuclear receptors and transcription factors, including peroxisome proliferator-activated receptor γ (PPARγ), forkhead box A1 (FOXA1), GATA binding protein 3 (GATA3), and E74 like ETS transcription factor 3 (ELF3), which are upstream of urothelial differentiation-restricted genes such as uroplakins and barrier-forming tight junction components.^16^, ^17^, ^18^ The tissue-restricted nature of the urothelial development program is illustrated by comparing urothelial with stratified squamous buccal epithelial-derived cells grown in identical conditions; buccal epithelial cells lack expression of key urothelial transcription factors and do not develop a transitional phenotype or barrier function under differentiation-inducing conditions.^19^

This study used the *in vitro* culture system described to examine whether epithelial cells isolated from a cohort of “delayed primary” or “secondary closure” BEX biopsy specimens are inherently squamous or transitional in phenotype, with genuine urothelial and buccal epithelial cells used as controls. The objectives of this study were to expand BEX-derived epithelial cells in culture and expose them to a permissive differentiation-inducing environment before assessing the signature profile of cytokeratin and transcription factor expression and the capacity to form a functional barrier measured as TEER.

## Materials and Methods

### Ethics and Consent

BEX bladder biopsy specimens were collected with appropriate parental consent and National Health Service Research Ethics Committee approval from Great Ormond Street Hospital, Evelina London Children's Hospital, and Manchester Royal Children's Hospital. Histology control specimens, including control buccal and tonsil tissue, were supplied by the National Health Service Research Ethics Committee–approved research tissue bank, URoBank, based in the Jack Birch Unit at the University of York (York, UK).

### Tissue Samples

Tissue samples were collected during 33 surgical procedures for BEEC; primary closure (*n* = 7), delayed primary closure (*n* = 6) or secondary procedure (*n* = 17), cloacal exstrophy (*n* = 2), or epispadias (*n* = 1). The male to female ratio in our complete cohort was 21 to 12. Control tissues were included of normal pediatric bladder, adult ureter, and buccal epithelium. Tissue details are shown in Supplemental Table S1.

A representative fraction of each tissue was fixed directly into 10% formalin for paraffin wax processing for (immuno)histology. The remainder sample was collected aseptically into transport medium comprising Hanks' balanced salt solution with 10 mmol/L HEPES pH 7.6 and 20 kallikrein inactivator units Trasylol (Bayer plc, Reading, UK). In addition, 2% penicillin-streptomycin (Merck, Poole, UK) was included because of the open, nonsterile nature of BEX bladders. Samples were transported on ice to the laboratory, where they were processed for cell culture within 48 hours.

### Histology and Immunohistochemistry

Tissue sections (5 μm) collected onto Superfrost Plus slides (Thermo Fisher Scientific, Waltham, MA) were dewaxed, rehydrated, and stained with hematoxylin and eosin for histologic assessment.

For immunohistochemistry, rehydrated sections were blocked for endogenous peroxidase activity before performing antigen retrieval as appropriate to the primary antibody (Table 1). For heat-induced epitope retrieval, slides were submerged in either 10 mmol/L citric acid buffer (pH 6.0) or 1 mmol/L Tris-EDTA buffer (pH 9.0), and heated in a pressure cooker for 10 minutes at 50 kPa. For CK14 labeling, slides were incubated for 60 seconds in 0.1% trypsin in 0.1% calcium chloride at 37°C before performing heat-induced epitope retrieval.

**Table 1:** Antibodies

Target antigen	Antibody name	Host	Antigen retrieval/detection method	Dilution	Source (catalog number)
CK14	LL002	Mouse	Trypsin+HIER (CA)/ABC	1/4800	Bio-Rad Laboratories, Watford, UK (MCA890)
CK13	1C7	Mouse	HIER (CA)/polymer	1/100	Origene supplied by Cambridge Bioscience, Cambridge, UK (BM5047S)
Ki-67	MM1	Mouse	HIER (CA)/ABC	1/600	Leica Biosystems, Milton Keynes, UK (NCL-L-Ki67-MM1)
PPARγ	81B8	Rabbit	HIER (E)/polymer	1/1000	Cell Signaling Technology, Leiden, the Netherlands (2443)
GATA3	D13C9	Rabbit	HIER (CA)/polymer	1/800	Cell Signaling Technology (5852)
ELF3	HPA003479	Rabbit	HIER (CA)/polymer	1/1000	Atlas antibodies supplied by Cambridge Bioscience
ΔNp63	Poly6190	Rabbit	HIER (CA)/polymer	1/8000	BioLegend UK Ltd., London, UK
TAp63	Poly6189	Rabbit	HIER (CA)/polymer	1/2000	BioLegend

Antigen retrieval methods included heat-induced epitope retrieval (HIER), citric acid (CA), or EDTA (E) buffers. Immunohistochemistry detection used either the avidin-biotin complex (ABC) or polymer method, depending on the sensitivity required.

CK, cytokeratin; ELF3, E74 like ETS transcription factor 3; GATA3, GATA binding protein 3; PPARγ, peroxisome proliferator-activated receptor γ; TAp63, tumor protein p63 isoform with transactivation domain; ΔNp63, N-terminal truncated isoform of tumor protein p63.

Immunohistochemistry detection used either the avidin-biotin-peroxidase complex kit (Vectastain; Vector Laboratories, Peterborough, UK) or the ImmPRESS Excel Amplified HRP Polymer Staining Kit (Vector Laboratories) for more sensitive immunodetection.

Antibodies were optimized by titration and the use of appropriate positive and negative controls. After immunodetection, slides were counterstained in Mayer's hematoxylin before dehydration and mounting in DPX (VWR International Ltd., Lutterworth, UK). Slides were viewed on an Olympus BX60 microscope and digital micrographs were captured on a DP74 camera and Olympus CellSens (version 1.17) standard software (Olympus UK, Southend-on-Sea, UK).

### BEX Epithelial Cell Culture

After isolation and disaggregation, epithelial cells from BEX samples were maintained as serially propagated cell cultures as developed for NHU cells^20^ and applied to normal human buccal epithelial cells.^19^ Briefly, BEX biopsy specimens were incubated in 0.1% EDTA for 4 hours at 37°C to separate the epithelium from stroma. The epithelial cell sheets were incubated in 100 U/mL collagenase (Type IV; Sigma-Aldrich Co. Ltd., Gillingham, UK) for 20 minutes at 37°C, before disaggregating sheets by pipetting and seeding at 4 × 10^4^ cells/cm^2^ on Cell+ plasticware (Sarstedt Ltd., Leicester, UK) in keratinocyte serum-free medium containing bovine pituitary extract and epidermal growth factor (Invitrogen Ltd., Inchinnan, UK) plus 30 ng/mL cholera toxin (referred to as KSFMc).^20^ Where only low numbers of cells were recovered, primary cultures were seeded onto a lawn of irradiated 3T3 feeder cells to support initial growth.^21^ Cultures were passaged by trypsinization at just-confluence and maintained as serially passaged finite cell lines. Cultures did not exceed four passages during experimentation. Where irradiated 3T3 J2 feeders were used, they were seeded at 3 × 10^4^ cells/cm^2^ in Dulbecco’s modified Eagle medium with 10% newborn bovine serum for 4 hours, before replacing the culture medium with KSFMc and seeding with urothelial cells. Antibiotics were included in primary cultures if indicated by the patient history.

### Electrophysiology Studies

To test if BEX cultures would undergo differentiation to form tight urothelial barriers, cultures were induced to differentiate as described previously.^15^^,^^22^ Briefly, cell cultures were grown to 80% confluence and the medium was changed to contain 5% (vol/vol) adult bovine serum (SeraLab, Burgess Hill, UK) for 5 days. Cultures then were harvested by trypsinization and seeded at 5 × 10^5^ cells per ThinCert membrane (113 mm^2^, 0.4 μm pore size; Greiner Bio-One Ltd., Stonehouse, UK). After 24 hours, the medium was changed to contain 5% adult bovine serum and 2 mmol/L [Ca^2+^], with cultures maintained for a further 8 days. TEER measurements were taken on days 6 and 8 using chopstick STX2 electrodes and an epithelial voltohmmeter (World Precision Instruments, Hitchin, UK). Resistance (Ω.cm^2^) was derived from applying Ohm's law.

### Real-Time Reverse-Transcribed Quantitative PCR

At the end point of electrophysiology studies, cell cultures were harvested in TRIzol reagent and RNA extraction was performed using a phenol-chloroform method according to the manufacturer's instructions (Thermo Fisher Scientific). Any contaminating genomic DNA was removed by DNase digestion (DNA-free; Thermo Fisher Scientific). cDNA was synthesized from 1 μg total RNA using Oligo(dT) 12 to 18 primers (Thermo Fisher Scientific). Real-time reverse-transcribed quantitative PCR was performed as described previously.^16^ A list of PCR primers is shown in Table 2.

**Table 2:** Primers Used for Real-Time Reverse-Transcribed Quantitative PCR

Gene	Forward primer	Reverse primer
*ELF3*	5′-TCAACGAGGGCCTCATGAA-3′	5′-TCGGAGCGCAGGAACTTG-3′
*FOXA1*	5′-CAAGAGTTGCTTGACCGAAAGTT-3′	5′-TGTTCCCAGGGCCATCTGT-3′
*GATA3*	5′-TCTATCACAAAATGAACGGACAGAA-3′	5′-TGTGGTTGTGGTGGTCTGACA-3′
*KRT13*	5′-TGTTGACTTTGGTGCTTGTGATG-3′	5′-GTTCTGCATGGTGATCTTCTCATT-3′
*KRT14*	5′-CGGCCTGCTGAGATCAAAGA-3′	5′-ATCTGCAGAAGGACATTGGCA-3′
*PPARG*	5′-GAACAGATCCAGTGGTTGCAG-3′	5′-CAGGCTCCACTTTGATTGCAC-3′
*GAPDH*	5′-CAAGGTCATCCATGACAACTTTG-3′	5′-GGGCCATCCACAGTCTTCTG-3′

### Statistical Analysis

For comparisons between squamous and transitional samples, unpaired two-tailed *t*-tests were performed using GraphPad Prism 8.3.0 (San Diego, CA).

## Results

### Histology of BEX Samples

Hematoxylin and eosin staining was performed to assess the morphology of the epithelium from BEX bladder samples. Nineteen of the 33 samples showed a squamous morphology (15 squamous and 4 mixed squamous/transitional), including 6 (of 7) samples taken at primary newborn closure and 4 (of 5) samples from delayed primary closure (Supplemental Table S1). The remaining 14 samples showed a transitional morphology (confirmed by histopathologist J.St.).

### *In Situ* Differentiation and Proliferation Status of BEX Samples

**Figure 1:**
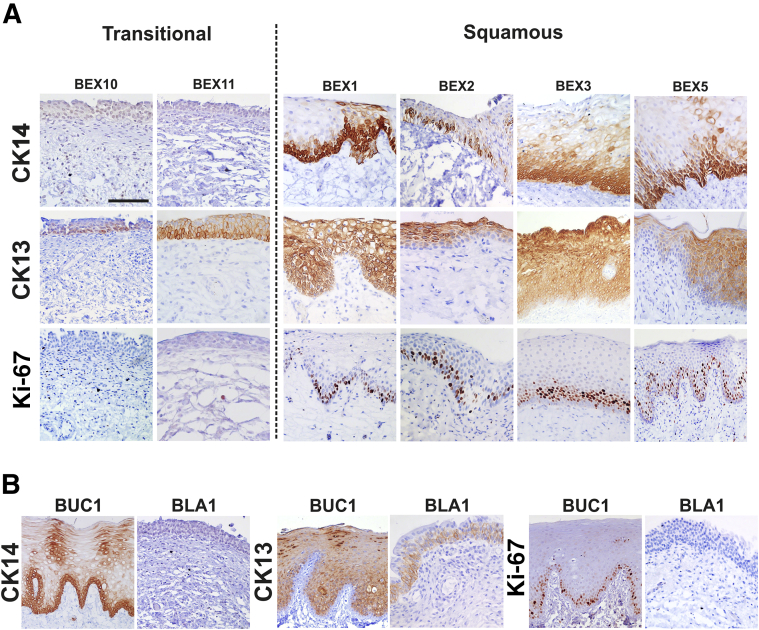
*In situ* expression (immunohistochemistry) of cytokeratin (CK)14, CK13, and Ki-67 in bladder exstrophy (BEX). **A:** Immunohistochemical labeling of CK14, CK13, and the cell-cycle marker Ki-67 in representative BEX biopsy specimens with transitional and squamous epithelia. Note: two representative transitional samples are shown, but because of their labeling variability, four squamous samples are shown. **B:** Control normal bladder (BLA1) and buccal (BUC1) tissues are included for comparison. Scale bar = 100 μm.

Immunohistochemistry was performed on the tissues from a subset of 11 BEX samples (Table 3), labeling for CK13 and CK14 and the cell-cycle marker Ki-67. Representative images are shown in Figure 1.

**Table 3:** Patient Samples Assessed by Immunohistochemistry and *in Vitro*

Patient number	Tissue type	Age	Sex	Surgical procedure
BEX1	BEX	5 months	M	Primary delayed closure
BEX2	BEX	19 months	M	Secondary procedure (Kelly surgery)
BEX3	BEX	84 months	F	Secondary procedure (bladder augmentation)
BEX4	BEX polyp cloacal exstrophy	1.3 months	M	Polypectomy (delayed closure)
BEX5	BEX	4 months	M	Primary delayed closure
BEX6	BEX bladder neck	204 months	F	Secondary procedure (bladder augmentation)
BEX7	BEX	6 months	M	Secondary procedure (Kelly surgery)
BEX8	BEX	42 months	M	Secondary procedure (Kelly surgery)
BEX9	BEX (epispadias bladder)	10 months	F	Secondary procedure (Kelly surgery)
BEX10	BEX	8 months	F	Secondary procedure (Kelly surgery)
BEX11	BEX	25 months	M	Secondary procedure (Kelly surgery)
CON1	Ureter	–	M	Renal transplant
CON2	Bladder	4 years	M	Ureteric reimplantation
BUC1	Buccal	46 years	M	Urethroplasty

F, female; M, male; BEX, bladder exstrophy; BUC, Buccal; CON, normal tissue control.

BEX bladders identified as transitional from hematoxylin and eosin staining (BEX6 to BEX11) were negative for the squamous marker CK14 by immunohistochemistry. All squamous BEX samples (BEX1 to BEX5) were CK14-positive (Figure 1 and Table 4). In the squamous samples, CK14 labeling was restricted to either basal or basal/intermediate cell layers. Buccal epithelium used as an internal positive control has a squamous morphology and CK14 was observed in basal/intermediate cell layers. Control bladder urothelium was negative for CK14.

**Table 4:** Summary of Immunohistochemistry Labeling of BEX Samples

Sample ID	CK14	CK13	Ki-67	PPARγ	GATA3	ELF3	ΔNp63	TAp63	TEER, Ω.cm^2^
BEX1	B	FT	B	(FT)	–	FT	B/I	(O)	621.5
BEX2	B	SB	B/I	–	–	FT	FT	–	128.1
BEX3	B/I	FT	I	(FT)	(O)	FT	B/I	(O)	2036.8
BEX4∗	B/I	FT	B	(FT)	–	FT	B/I	(O)	79.1
BEX5	B	SB	B/I	(S)	(O)	FT	B/I	(O)	2440.8
BEX6	–	B	O	FT	FT	FT	B/I	(O)	1956.3
BEX7	–	B/I	O	FT	FT	FT	B/I	–	1628.0
BEX8	–	B/I	O	FT	FT	FT	B/I	(O)	1252.8
BEX9	–	B/I	–	FT	FT	FT	B/I	(O)	2092.4
BEX10	–	B/I	–	FT	FT	FT	FT	(O)	3909.8
BEX11	–	B/I	O	FT	FT	FT	FT	(O)	1140.2
BLA1	–	B	–	FT	FT	FT	FT	–	NA
BUC1	B/I	SB	B	–	(B)	–	B/I	–	NA

**Figure 2:**
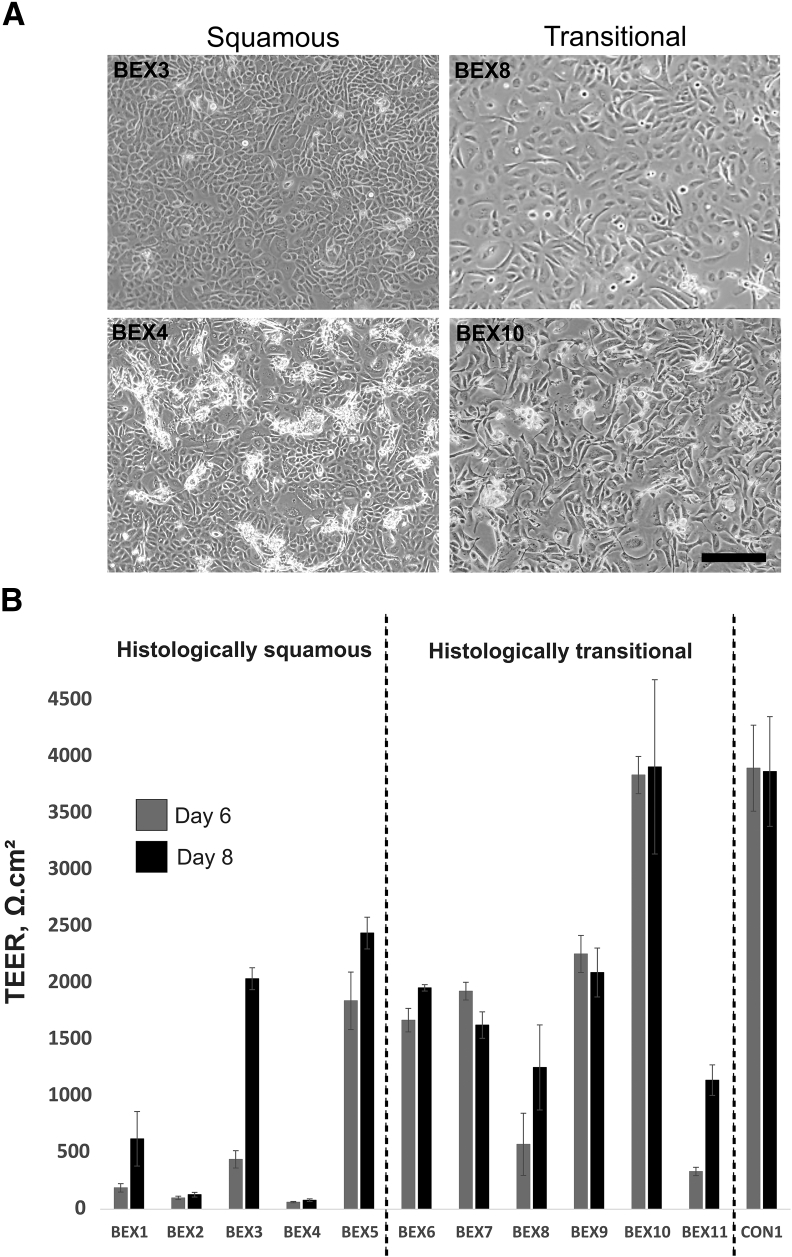
Bladder exstrophy (BEX) cell cultures and measurement of barrier function. **A:** Representative phase-contrast images of BEX cells derived from histologically squamous or transitional samples after growth *in vitro*. **B:** Transepithelial electrical resistance (TEER) measurements from BEX cultures from 11 donor patients on days 6 and 8 after switching to differentiation-inducing medium. BEX samples were divided into those derived from squamous or transitional epithelial samples based on the original histologic assessment. A TEER ≥1000 Ω.cm^2^ was regarded as the standard for defining a tight epithelial^15^^,^^19^ barrier. **B:** Means ± SD; *n* = 4 to 6 replicates. Scale bar = 200 μm. CON1, normal human ureteric cells included as control.

Immunohistochemical labeling of the BEX study cohort (BEX1 to BEX11) with CK14, CK13, Ki-67, peroxisome proliferator-activated receptor γ, GATA3, ELF3, ΔNp63, and TAp63. Control normal bladder (BLA1) and buccal (BUC1) tissues were included. Mean TEER measurements (*n* = 4 to 6 replicates) are shown (from day 8) (Figure 2). Note that 1000 Ω.cm^2^ is used as the cut-off value for a tight barrier. Parentheses indicate weak-intensity labeling.

B, basal; BEX, bladder exstrophy; CK, cytokeratin; ELF3, E74 like ETS transcription factor 3; FT, full thickness; GATA3, GATA binding protein 3; I, intermediate layers; NA, not applicable; O, occasional positive; S, superficial; SB, suprabasal; TAp63, tumor protein p63 isoform with transactivation domain; TEER, transepithelial electrical resistance; ΔNp63, N-terminal truncated isoform of tumor protein p63; –, negative.

∗ Sample taken from polyp.

CK13 is characteristically expressed by basal and intermediate, but not superficial cells in urothelium, whereas it is excluded from basal cells in stratified squamous epithelia.^23^ Normal human bladder urothelium expressed CK13 in the basal and intermediate cell layers (Figure 1B), and all BEX samples classified as transitional followed this basal/intermediate pattern of expression (Figure 1A and Table 4). Squamous BEX samples showed either full-thickness or suprabasal CK13 expression (Figure 1A and Table 4), the latter being equivalent to the expression pattern observed in buccal epithelium (Figure 1B).

In all 11 BEX samples, epithelial morphology (transitional or squamous) defined the pattern of Ki-67–positive cells. Transitional samples contained a low proportion of Ki-67–positive cells dispersed throughout the epithelium and characteristic of mitotically quiescent normal bladder urothelium. In squamous epithelia, Ki-67–positive cells were found in the basal and suprabasal layers across the full width of the epithelium, indicating cell-cycle activity; this expression was comparable with that observed in buccal epithelium (Figure 1 and Table 4).

### Cell Culture and Capacity for BEX-Derived Epithelial Cells to Form Differentiated Urothelium *in Vitro*

Epithelial cells isolated from primary delayed closure or secondary BEX samples categorized as squamous (*n* = 5) or transitional (*n* = 6) were established in culture. Patient details are shown in Table 3.

BEX cultures grew as proliferative monolayers in low-calcium, serum-free medium, forming contact-inhibited epithelioid pavement cultures at confluence. Cultures of cells isolated from squamous biopsy specimens were indistinguishable from those isolated from transitional biopsy specimens (Figure 2A). Using the protocol developed to differentiate NHU cell cultures, the ability of BEX cells to develop functional tight barriers was assessed in cultures seeded on permeable membrane inserts in medium supplemented with serum and 2 mmol/L calcium. After 8 days, all but 3 (of 11) BEX cultures were capable of forming a tight barrier greater than 1000 Ω.cm^2^ (Figure 2B). The three BEX samples that failed to develop tight barriers were derived from samples identified histologically as squamous. The ureteric NHU control culture formed a tight barrier and, as reported previously,^19^ buccal epithelial cell cultures did not.

### Phenotype of BEX Epithelial Cell Cultures

**Figure 3:**
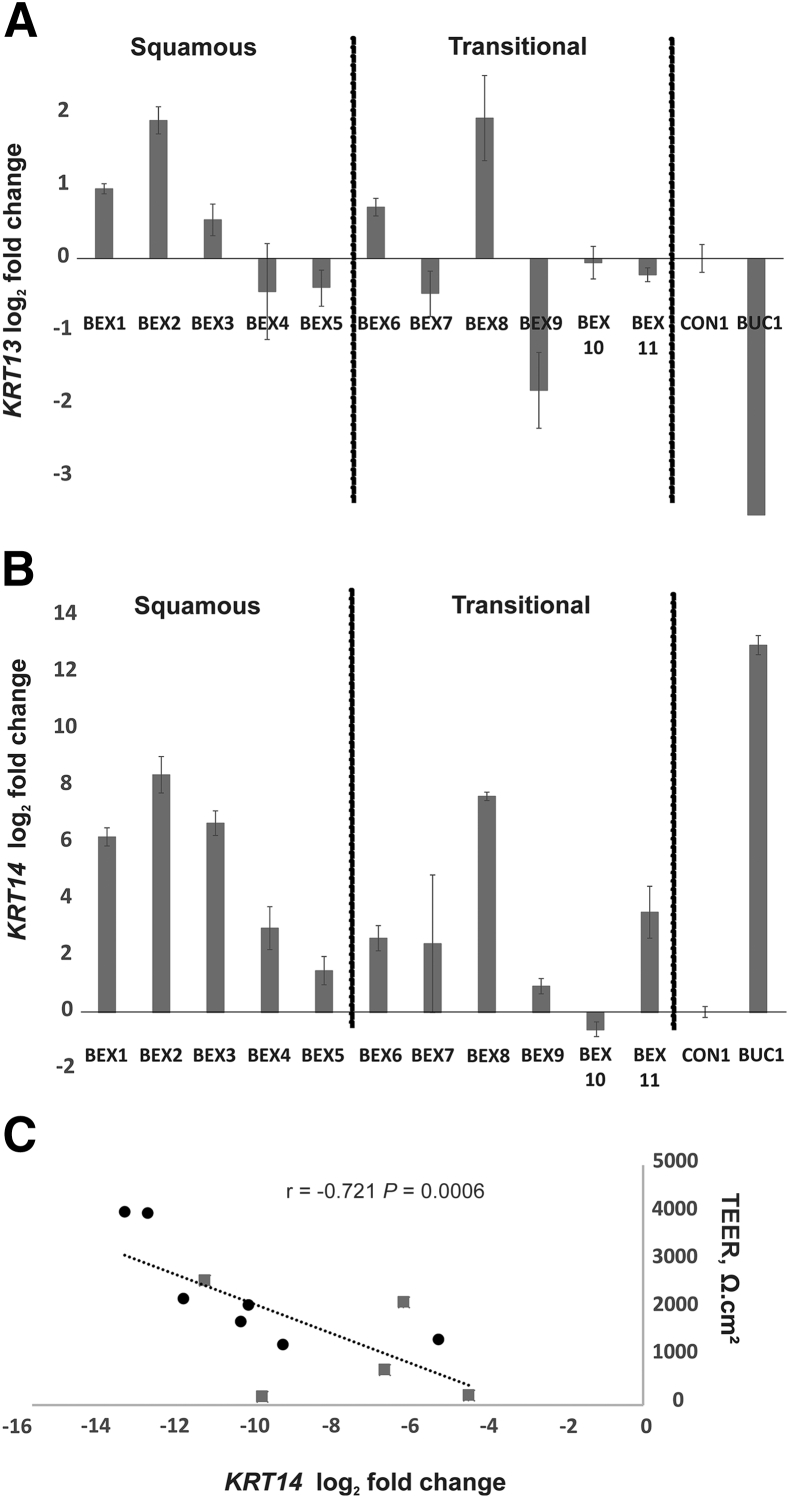
*KRT13* and *KRT14* transcript expression in bladder exstrophy (BEX) cultures. **A** and **B:** Real-time reverse-transcribed quantitative PCR analysis of BEX cell cultures harvested after transepithelial electrical resistance (TEER) analysis (day 8) for *KRT13* expression (**A**) or *KRT14* expression (**B**). BEX cultures were separated into histologically (*in situ*) squamous or transitional, as indicated. Expression is plotted as log_2_ fold change, relative to a ureteric normal human urothelial cell culture control (CON1). A human buccal control culture (BUC1) was included as a control for *KRT13* and *KRT14* expression. A two-tailed *t*-test was performed to assess whether expression in squamous samples was significantly different than transitional samples. **C:** Pearson correlation of *KRT14* gene expression with barrier function (TEER) on day 8 after differentiation (**gray boxes** indicate squamous samples, and **black circles** indicate transitional samples). The **diagonal line** shows the line of best fit. **A** and **B:***n* = 11.

Expression of *KRT13* and *KRT14* transcripts by BEX-derived epithelial cell cultures was examined by real-time reverse-transcribed quantitative PCR. After 8 days in urothelial differentiation–inducing conditions, all BEX cultures showed higher *KRT13* and lower *KRT14* expression than buccal epithelial cell cultures included as control. Comparing cultures that derived originally from histologically squamous versus transitional type BEX *in situ*, no significant difference was found in either the *in vitro* expression of *KRT13* (Figure 3A), or *KRT14* (Figure 3B). *KRT14* gene expression by BEX cultures showed a significant negative association with donor-matched TEER values (*P* = 0.0006) (Figure 3C), whereas no relationship was found between *KRT13* expression and TEER (*P* = 0.257; data not shown).

### Expression of Transcription Factors in BEX Cultures

**Figure 4:**
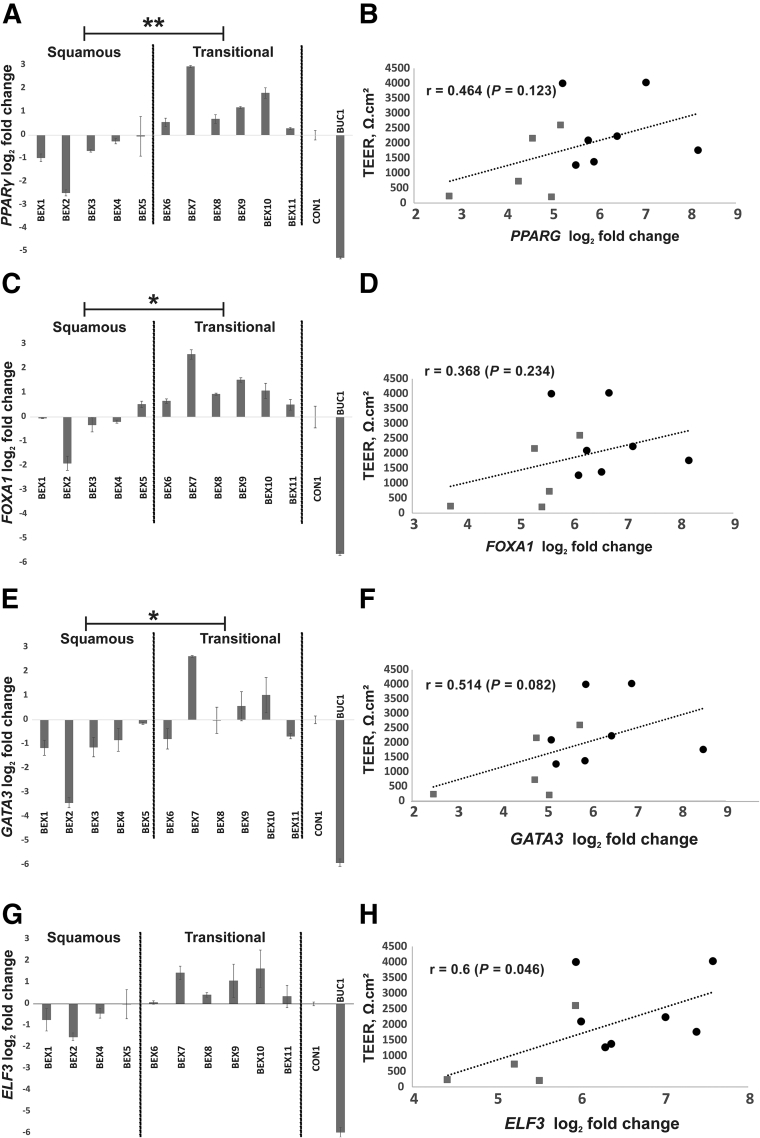
Transcript expression of urothelial differentiation-associated transcriptional regulators in bladder exstrophy (BEX) cultures. **A**, **C**, **E**, and **G:** Real-time reverse-transcribed quantitative PCR analysis of differentiated (day 8) BEX cell cultures for *PPARG* (**A**), *FOXA1* (**C**), *GATA3* (**E**), and *ELF3* (**G**) expression. Expression is plotted as log_2_ fold change, relative to control normal human urothelial cell culture (CON1). A human buccal epithelial cell culture was included (BUC1). **B**, **D**, **F**, and **H:** Pearson correlation of transcription factor gene expression with barrier function [transepithelial electrical resistance (TEER)] on day 8 after differentiation is shown. **Gray boxes** indicate histologically identified squamous samples, and **black circles** indicate transitional samples. The **diagonal line** shows the line of best fit. ∗*P* < 0.05, ∗∗*P* < 0.01 (two-tailed *t*-tests comparing delta cycle threshold values from transitional versus squamous sample groups).

Real-time reverse-transcribed quantitative PCR was used to evaluate expression of the transcriptional regulators *PPARG*, *FOXA1*, *GATA3*, and *ELF3* in BEX cultures after TEER analysis (day 8) (Figure 2). Significantly higher expression of *PPARG*, *FOXA1*, and *GATA3* was observed in BEX cultures derived from histologically transitional versus squamous epithelium (Figure 4, A, C, and E). Although not significant, *ELF3* transcript also was higher in transitional samples (Figure 4G). Expression of all four transcription factors was lower in control buccal cultures relative to either BEX or control NHU cell cultures. A positive (Pearson) correlation was found between *PPARG*, *FOXA1*, *GATA3*, and *ELF3* expression and barrier function (TEER), although only *ELF3* was significant (Figure 4, B, D, F, and H).

### Expression of Urothelial Transcription Factors *in Situ*

To examine whether the *in vitro* transcription factor expression profile matched the original *in situ* expression pattern and correlated to *in situ* differentiation status, expression of PPARγ and urothelial transcription factors was examined by immunohistochemistry. These results are summarized in Table 4.

**Figure 5:**
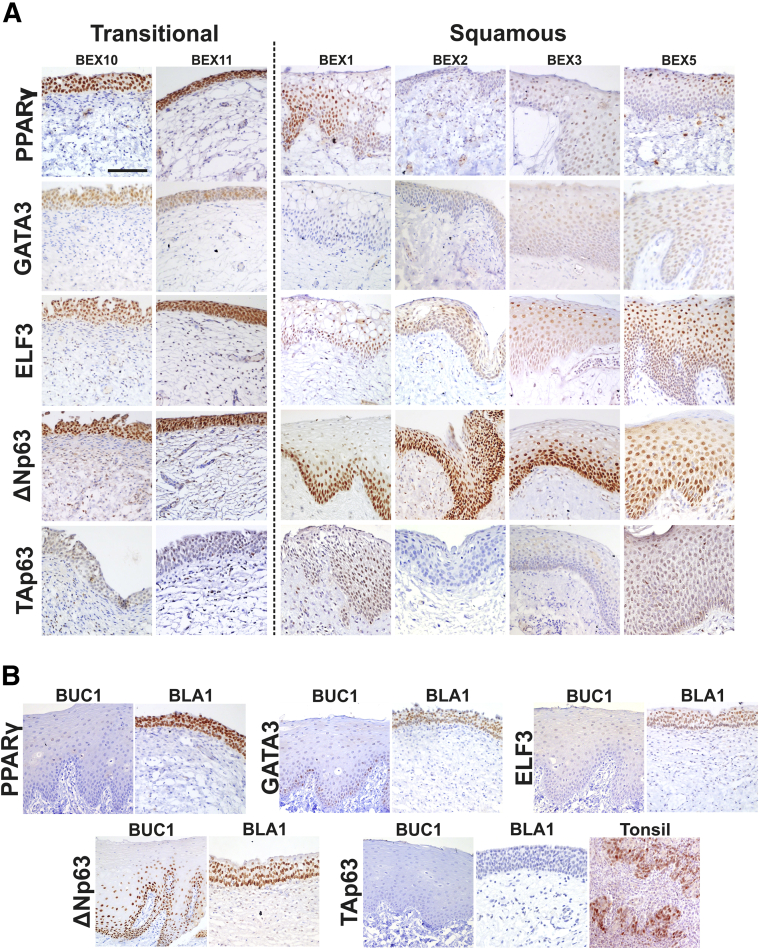
*In situ* expression of urothelial differentiation–associated transcription factors. **A:** Immunohistochemical labeling of peroxisome proliferator-activated receptor γ (PPARγ), GATA binding protein 3 (GATA3), E74 like ETS transcription factor 3 (ELF3), N-terminal truncated isoform of tumor protein p63 (ΔNp63), and tumor protein p63 isoform with transactivation domain (TAp63) in representative bladder exstrophy (BEX) biopsy specimens with transitional or squamous habit. Control normal bladder (BLA1) and buccal (BUC1) tissues are included for comparison. **B:** Tonsil tissue is included as control for TAp63 owing to the lack of expression in buccal or bladder epithelia. Scale bar = 100 μm.

All BEX biopsy specimens that contained transitional urothelium showed full-thickness, intense nuclear PPARγ labeling (BEX6 to BEX11), comparable with normal bladder control urothelium (Table 4 and Figure 5). Squamous BEX samples (BEX1 to BEX5) contained weaker and more variable PPARγ labeling than transitional samples. In three of five squamous BEX samples, the PPARγ labeling was full thickness but weak, another contained only superficial PPARγ labeling, while the remaining squamous BEX sample was negative for PPARγ. Buccal epithelium was negative for PPARγ *in situ*.

Urothelium from transitional BEX samples (BEX6 to BEX11) contained strong, full-thickness nuclear GATA3 labeling, comparable with control bladder. Of the five squamous BEX samples studied, three were negative for GATA3, with the remaining samples containing weak occasional positive nuclei in the epithelium (Table 4 and Figure 5). All three squamous BEX samples lacking GATA3 expression *in situ* failed *in vitro* to generate a tight TEER (Table 4). Buccal epithelium was primarily negative for GATA3 expression, but two of three buccal control specimens contained rare, extremely weak, GATA3-positive nuclei in the basal cell layer only (Figure 5B).

ELF3 expression was present in all cell layers in the epithelium of all 11 BEX biopsy specimens, regardless of squamous/transitional phenotype (Table 4 and Figure 5A). Strong, full-thickness labeling also was observed in the control bladder epithelium, whereas buccal epithelium was negative for ELF3 *in situ* (Figure 5B).

The ΔNp63 isoform was the predominant p63 form in bladder urothelium, as well as all 11 BEX biopsy specimens tested. Expression of ΔNp63 was intensely basal/intermediate in four of five squamous BEX samples and full thickness in the remaining squamous sample. In transitional samples, four of six showed intense basal/intermediate labeling, while two of six showed full thickness as in the control bladder urothelium (Table 4 and Figure 5). Expression of TAp63 was very weak in BEX samples, with occasional positive nuclei in four of five squamous and five of six transitional samples. TAp63 was absent in control bladder urothelium. In buccal epithelium, ΔNp63 was strongly expressed in the basal and intermediate cell layers, however, TAp63 was absent (Table 4 and Figure 5).

## Discussion

This study isolated the presumptive urothelial cells from bladder exstrophy tissues from a small cohort of infants with the aim of determining their phenotype and inherent capacity to form a functional barrier urothelium. Histologic and immunohistochemical analysis was used to classify the *in situ* phenotype as squamous or transitional, in agreement with previous findings that squamous metaplasia is a common histologic feature of the exstrophy bladder.^11^ Although the numbers were small, no particular pattern of squamous or transitional differentiation was observed relating to whether samples were collected at primary closure, after a delayed closure to allow for growth of the bladder plate, or at the time of secondary reconstructive surgery. This supports the conclusions made by other investigators that histologic changes in the exstrophy bladder persist beyond primary closure.^12^^,^^24^ However, our approach enabled a more nuanced stratification of the squamous samples, in which three of five remained intractably squamous, but two of five underwent transitional differentiation with concomitant barrier formation *in vitro*. Given that the primary function of the urothelium is to act as a barrier to protect underlying tissues from the damaging effects of urine, the distinction between reversible or intractable squamous differentiation could carry critical implications both for predicting bladder function after closure and for selecting patients for new tissue-engineered strategies using autologous cells.

Under identical *in vitro* conditions, human urothelial^15^ and buccal epithelial^19^ cells have been shown to lose differentiated features to form very similar proliferative epithelioid monolayers. However, each retains their specified differentiation program in response to permissive factors in serum, with only urothelial cells forming a tight barrier (>1000 Ω.cm^2^). It was hypothesized that human urothelial and buccal cells retain a programed epigenetic memory *in vitro*.^19^ Herein, this well-characterized cell culture system was used to isolate and subculture epithelial cells from histologically transitional and squamous BEX samples. For cells isolated from BEX bladders there is added complexity in interpreting whether any aberrant differentiation is genetic in cause, results from failure of instructive epigenetic programming during differentiation, or is secondary to long-term exposure with an open environment. BEX cells cultured from transitional samples showed the capacity to develop a functional barrier measured by TEER, but although two of five squamous-derived cultures also were able to form a barrier, three of five were impaired, including one squamous sample in which the cells were isolated from a polyp. We surmise that isolation of BEX cells from samples with transitional histology confers barrier-forming ability, but that there is more variability among the histologically squamous BEX samples.

To consider the molecular basis for differences between transitional and squamous phenotypes, transcript expression of the archetypal urothelial transcriptional regulators *PPARG*, *FOXA1*, and *GATA3* was examined and found to be significantly lower in squamous than in transitional BEX epithelial cell cultures. The squamous BEX samples also showed relatively lower *in situ* expression of PPARγ and GATA3 by immunohistochemistry. Although the numbers are small, absent GATA3 immunoexpression was noted to be consistent with a failure to form a functional barrier when the counterpart cells were differentiated *in vitro*, suggesting GATA3 expression segregated the reversible from intractable squamous BEX subgroups. PPARγ is a regulator of the urothelial transitional differentiation program,^14^^,^^25^^,^^26^ and, acting downstream of activated PPARγ, GATA3 represses the nondifferentiated squamous/basal phenotype mediated by p63.^18^ In muscle-invasive bladder cancer, *TP63* is reported to be a driver of the aggressive basal/squamous gene signature by up-regulating *KRT5* and *KRT14*,^5^^,^^27^ and in other tissues *KRT14* has been shown to be a direct transcriptional target of ΔNp63α.^28^^,^^29^ Although no differences in p63 expression between transitional and squamous samples were observed by immunohistochemistry, the absence of p63 repression by GATA3 may provide a determinant for squamous differentiation.

*In situ* buccal epithelium expressed ΔNp63, which was also present in all BEX samples, regardless of morphology. Mechanistically, ΔNp63 is complex and involved in both activation and repression of gene transcription by enhancement of chromatin accessibility or interaction with epigenetic repressors, respectively.^30^ Although the current study adds further weight to the dominant function of ΔNp63, rather than the TAp63 isoform in urothelium, we conclude that the epigenetic landscape in which BEX urothelium presents is likely to play a key role in determining the ability of p63 to drive a more transitional or squamous phenotype.

*In situ*, squamous BEX samples were more comparable with other stratified squamous epithelia than urothelium, with full-thickness CK13 expression, basal CK14 expression, and basal/intermediate Ki-67 labeling, comparable with normal buccal and cervical epithelia.^19^^,^^31^^,^^32^ This indicates that squamous BEX epithelium has a constitutive rate of cell turnover, whereas transitional-type BEX showed only rare dispersed Ki-67–positive nuclei, in line with healthy mitotically quiescent urothelium.^33^ This is of particular interest because there is a potential for an epithelium with constant turnover and compromised urinary barrier to be more susceptible to carcinogenic agents from exposure to urine. Although the current results indicate the potential for some histologically squamous BEX epithelia to revert to a barrier-forming transitional phenotype under permissive conditions, it raises a particular concern that intractable squamous BEX samples may be fundamentally incapable of generating a mitotically quiescent urinary barrier. Given that several clinical centers have BEX histology collections, it is worth considering a retrospective study to stratify squamous BEX samples on the basis of GATA3 expression and relate it to long-term patient outcomes.

Primary bladder closure, continence procedures, and augmentation enterocystoplasty are the most common surgical interventions adopted for BEX management. Mid- and long-term complications after enterocystoplasty include recurrent infections and possible malignancy resulting from long-term exposure of the non–barrier-forming and absorptive intestinal epithelium to urine.^34^ When new approaches are being developed, such as enlarging the bladder using autologous cells,^35^ understanding the BEX epithelial phenotype could help in selecting the most appropriate patients. From the work presented here, histologically transitional BEX carry the best potential to be propagated *in vitro* and develop into a functional barrier-forming urothelium for tissue-engineering applications. Although the intractable squamous BEX would be considered unsuitable, this study identified a subset of squamous presenting BEX that were able to be expanded and differentiated to form a urothelial barrier *in vitro*. This suggests that there may be some factor in the *in situ* BEX tissue environment that inhibits some epithelia from showing their urothelial potential. Further research aimed, for example, at understanding the drivers of GATA3 expression may provide additional insight.

The etiology of BEEC is still not fully understood but better stratification of BEX epithelial subsets should help inform future management and treatment strategies. Moreover, the information provided by this study will help design future therapeutic strategies based on tissue-engineering technologies for bladder augmentation. Based on the current results, stratifying BEX patients into one of three groups on the basis of our histologic findings is recommended, as follows: i) consistently transitional, with high expression of PPARγ/GATA3, low expression of Ki-67, and CK14 negative; ii) squamous, but with barrier-forming potential, with high expression of CK14 and Ki-67 (basal) and low PPARγ and GATA3 expression; and iii) intractably squamous, characterized by a complete lack of GATA3 expression, high expression of CK14 and Ki-67 (basal), and low PPARγ.
