# Supplementary material for: Barrier-Forming Potential of Epithelial Cells from the Exstrophic Bladder
Source: Am J Pathol. 2022 Jun;192(6):943–55. doi: 10.1016/j.ajpath.2022.03.009 (PMC9194657; doi:10.1016/j.ajpath.2022.03.009)
Supplement: Supplemental Table S1 [file mmc1.docx]

|  | **Male** samples | **Female** samples | **Median age** in months (range) | **Histology**  **Sq/T/Mix** |
| --- | --- | --- | --- | --- |
| **Bladder exstrophy**  **Primary**  *Bladder*  *Polyps*  **Delayed closure**  *Bladder*  *Polyps*  **Secondary**  *Bladder*  *Polyps*  **Cloacal exstrophy**  **Epispadias** | 2  3  -  3  11  1  1  - | 2  -  1  1  6  -  1  1 | 0.55 (0.1-4)  5 (0.3-6)  7  7.5 (5-12)  24 (6-204)  10  24.65 (1.3-48)  10 | Sq(2), T(1) Mix(1)  Sq(1), Mix(2)  Sq(1)  Sq(2), T(1), Mix(1)  Sq(6), T(11)  Sq(1)  Sq(2)  T(1) |
| **Control Tissues**  Control ureter  Control bladder  Control buccal | 1  1  1 | -  -  - | Unknown  51  552 | T  T  S |

**Supplementary Table 1 – Patient-related data and epithelial histological findings.** BEX patients were split into three groups 1) primary closure (closure of the abdominal wall shortly after birth), 2) delayed closure (primary closure after growth of the bladder plate) and 3) secondary (reconstructive surgery after closure). Cloacal exstrophy and epispadias samples were also included. Control samples are also shown. Histology indicates the number of samples in which the epithelium was squamous (Sq), Transitional (T) or of mixed squamous/transitional morphology (Mix).
